# Evaluation of the immunogenicity of aldhhigh human head and neck squamous cell carcinoma cancer stem cells *in vitro*

**DOI:** 10.1186/2051-1426-3-S2-P28

**Published:** 2015-11-04

**Authors:** Qiao Li, Mark Prince

**Affiliations:** 1 University of Michigan Medical Center, Ann Arbor, MI, USA; 2 University of Michigan, Ann Arbor, MI, USA

## Background

Using mouse models we reported that dendritic cells (DC) pulsed with cancer stem cells (CSC) enriched by virtue of their expression of the CSC marker aldehyde dehydrogenase (ALDH) significantly reduced development of pulmonary metastases and prolonged survival. In this recent study, we established the concept that the antigenicity/immunogenicity of ALDH^high^ human head and neck squamous cell carcinoma (HNSCC) cancer stem cells is distinct from that of ALDH^low^ non-CSCs.

## Methods

Patients with HNSCC enrolled in the University of Michigan Special Project of Research Excellence (SPORE) were recruited to collect tumor and peripheral blood samples. T or B cells were purified from the PBMCs using anti-CD3-coupled or anti-CD19-coupled microbeads respectively with a MACS separator. Primary tumor samples were digested and collected. ALDEFLUOR^+^/ALDH^high^ or ALDEFLUOR^-^/ALDH^low^ cells were isolated from tumor cells. ALDH^low^ lysate-pulsed DCs (ALDH^low^ -DC) or ALDH^high^ lysate-pulsed DCs (ALDH^high^-DC, e.g. CSC-DC) were used as vaccines. To detect T and B cells in PBMCs, PBMCs were stained with PerCP mouse anti-human CD3 and FITC mouse anti-human CD19. Before sensitization *in vitro*, T or B cells were activated respectively with immobilized anti-human CD3 and anti-human CD28 in complete medium (CM) containing hrIL-2 or lipopolysaccharide plus anti-human CD45. Statistical analyses were performed to compare various interactions of the DC vaccine-primed/sensitized T, B cells with autologous ALDH^high^ CSC *vs*. ALDH^low^ HNSCC target cells.

## Results

DCs generated from the PBMC and pulsed with the lysate of ALDH^high^ cells isolated from cultured HNSCC cells (CSC-DC) could sensitize autologous T, B lymphocytes *in vitro*, which was evident by cytokine production, CTL activity, and antibody secretion of these primed T, B cells in response to ALDH^high^ CSCs. In contrast, DCs pulsed with lysate of ALDH^low^ cells from the same HNSCC patient (ALDH^low^-DC) resulted in limited sensitization/priming of autologous T, B lymphocytes to produce IFNg, lyse CSCs, and secrete IgM and IgG in response to ALDH^high^ CSCs.

## Conclusions

These results demonstrated significant differences in the antigenicity/immunogenicity between ALDH^high^ CSCs *vs*. ALDH^low^ cells isolated from the tumor specimen of patients with HNSCC, which indicates the existence of unique CSC antigens in the ALDH^high^ population. In addition, this study demonstrates that it is feasible to generate DCs from the PBMCs and isolate ALDH^high^ CSCs from tumor cells of the patients with HNSCC to prepare CSC-DC vaccines for clinical application.

